# MISSING VALUE IMPUTATION VIA GRAPH COMPLETION IN QUESTIONNAIRE SCORES FROM PERSONS WITH DEMENTIA

**DOI:** 10.1093/geroni/igz038.3524

**Published:** 2019-11-08

**Authors:** Chen Kan, Won Hwa Kim, Ling Xu, Noelle L Fields

**Affiliations:** University of Texas at Arlington, Arlington, Texas, United States

## Abstract

Background: Questionnaires are widely used to evaluate cognitive functions, depression, and loneliness of persons with dementia (PWDs). Successful assessment and treatment of dementia hinge on effective analysis of PWDs’ answers. However, many studies, especially pilot ones, are with small sample sizes. Further, most of them contain missing data as PWDs skip some study sessions due to their clinical conditions. Conventional imputation strategies are not well-suited as bias will be introduced because of insufficient samples. Method: A novel machine learning framework was developed based on harmonic analysis on graphs to robustly handle missing values. Participants were first embedded as nodes in the graph with edges derived by their similarities based on demographic information, activities of daily living, etc. Then, questionnaire scores with missing values were regarded as a function on the nodes, and they were estimated based on spectral analysis of the graph with a smoothness constraint. The proposed approach was evaluated using data from our pilot study of dementia subjects (N=15) with 15% data missing. Result: A few complete variables (binary or ordinal) were available for all participants. For each variable, we randomly removed 5 scores to mimic missing values. With our approach, we could recover all missing values with 90% accuracy on average. We were also able to impute the actual missing values in the dataset within reasonable ranges. Conclusion: Our proposed approach imputes missing values with high accuracy despite the small sample size. The proposed approach will significantly boost statistical power of various small-scale studies with missing data.

